# Laparoscopic Adrenal Surgery in an Adolescent Boy in the Caribbean With Malignant Hypertension Secondary to Pheochromocytoma: A Case Report and Literature Review

**DOI:** 10.7759/cureus.86623

**Published:** 2025-06-23

**Authors:** Shariful Islam, Joshua Ramoutar, Malini Ramnarine, Raphael Ramadan, Dilip Dan

**Affiliations:** 1 Department of Surgery, San Fernando General Hospital, San Fernando, TTO; 2 Department of Clinical Surgical Sciences, The University of the West Indies, St. Augustine, TTO; 3 Department of General Surgery, San Fernando General Hospital, San Fernando, TTO; 4 Department of Surgery/Minimally Invasive Surgery, San Fernando General Hospital, San Fernando, TTO

**Keywords:** hypertension in adolescents, hypertensive emergency, hypertensive urgency, laparoscopic adrenalectomy, malignant hypertension, pheochromocytoma

## Abstract

Malignant hypertension is defined as extremely elevated blood pressure (BP) with or without target organ damage. It can be either hypertensive urgency, characterized as malignant hypertension without any clear evidence of immediate organ damage, or hypertensive emergency with proof of new or worsening target-organ damage. The modern definition of hypertensive emergencies without retinopathy would be based on the presence of acutely raised BP with injury to at least three target organs. Pheochromocytoma is the rarest cause of malignant arterial hypertension, characterized by the classic triad of headache, sweating, and palpitations. It is rare in adolescents, and it often presents a unique surgical challenge due to catecholamine secretion. Laparoscopic adrenalectomy is the recommended surgical treatment of choice. Although it is a common procedure performed in adults, it has not yet been reported in adolescents in the Caribbean. This case report details the presentation of a 16-year-old boy from Trinidad and Tobago, who had a six-month history of episodic headaches, sweating, palpitations, blurry vision, and significantly elevated BP (up to 225/120 mmHg). His clinical picture was further complicated by new-onset diabetes mellitus and a family history of hypertension and diabetes on both maternal and paternal sides. Investigations revealed significantly elevated catecholamine levels, and imaging confirmed a right adrenal mass consistent with a pheochromocytoma. This case highlights both the clinical complexity and the feasibility of performing laparoscopic adrenalectomy in adolescent patients within a Caribbean healthcare setting.

## Introduction

Pheochromocytoma is a rare neoplasm of the chromaffin cells of the adrenal gland or other para-ganglia, occurring at an incidence of 0.6/100,000 [1]. These tumors secrete catecholamines and present unique challenges in surgical management. The minority of these tumors, 0.2 - 0.5/1,000,000, occur in the adolescent population and are more likely to be familial than sporadic [2]. Clinically, pheochromocytoma is characterized by paroxysmal or sustained hypertension, along with the classic triad of headaches, palpitations, and diaphoresis. Additional symptoms may include anxiety, weight loss, pallor, and visual disturbances. Biochemically, the diagnosis is confirmed through elevated plasma or urinary metanephrines and normetanephrines. Radiologic localization is typically achieved using contrast-enhanced CT (computed tomography) or MRI (magnetic resonance imaging), or PET-CT (positron emission tomography-CT) reserved for extra-adrenal or metastatic disease. Laparoscopic adrenalectomy remains the recommended surgical management for pheochromocytoma occurring in the adrenal gland [3]. Due to the rarity of this tumor, especially in young patients, and the infrequency of cases managed and documented in the Caribbean, the authors saw fit to report on a successful case managed in the public healthcare system of Trinidad and Tobago. 

## Case presentation

A 16-year-old boy with a BMI of 24.3 kg/m^2^ was referred from the endocrinology outpatient clinic to the general surgery service with the diagnosis of secondary hypertension due to a unilateral pheochromocytoma and diabetes mellitus. History revealed that the patient initially presented to the endocrine clinic with episodic headaches, diaphoresis, palpitations, blurry vision, and elevated blood pressure (BP) for six months. The patient also gave a maternal and paternal history of essential hypertension and diabetes mellitus. He was admitted to the medical ward for an episode of hypertensive emergency with a BP of 225/120 mmHg.

On this admission, he was managed with intravenous hypertensive medications with partial relief of symptoms. A detailed physical examination revealed no abdominal or neck masses, and an ophthalmoscopy examination revealed no abnormalities. During this admission, the patient was also found to be diabetic with an HbA1c of 8.9% and started on metformin. The patient was then referred to an endocrinologist for further workup.

Detailed blood investigations were within normal ranges, including a complete blood count, renal function tests, liver function tests, thyroid function tests, parathyroid hormone, and calcium. However, serum norepinephrine and normetanephrine levels were significantly elevated, i.e., 2279 (0-874) pg/mL and 2233.8 (0-150) pg/mL, respectively (Table 1). A diagnosis of pheochromocytoma was made, and a combination of antihypertensive medications, including amlodipine, enalapril, and terazosin, was prescribed.

**Table 1:** Summary of laboratory investigation results Bold values indicate significant findings

Category		Test	Result	Reference Value
Serum	Catecholamine	Norepinephrine	2279	0-874 pg/ml
		Epinephrine	50	0-62 pg/ml
		Dopamine	<30	0-48 pg/ml
		Normetanephrine	2233.8	0-150.8 pg/ml
		Metanephrine	25.2	0-88 pg/ml
Serum		Cortisol AM (low dose dexamethasone)	1.12	6.02-18.4 µg/d
Serum	Pre-Surgery	HBA1C	8.9 %	< 5.7 %
	Post-Surgery	HBA1C (%)	5.2 %	< 5.7%
Urine		Albumin	46.1	24-392 mg/dl
		Creatinine	106.43	22-328 mg/dl
		Albumin/creatinine ratio	43.3	0-30 µg/mg

**Figure 1:**
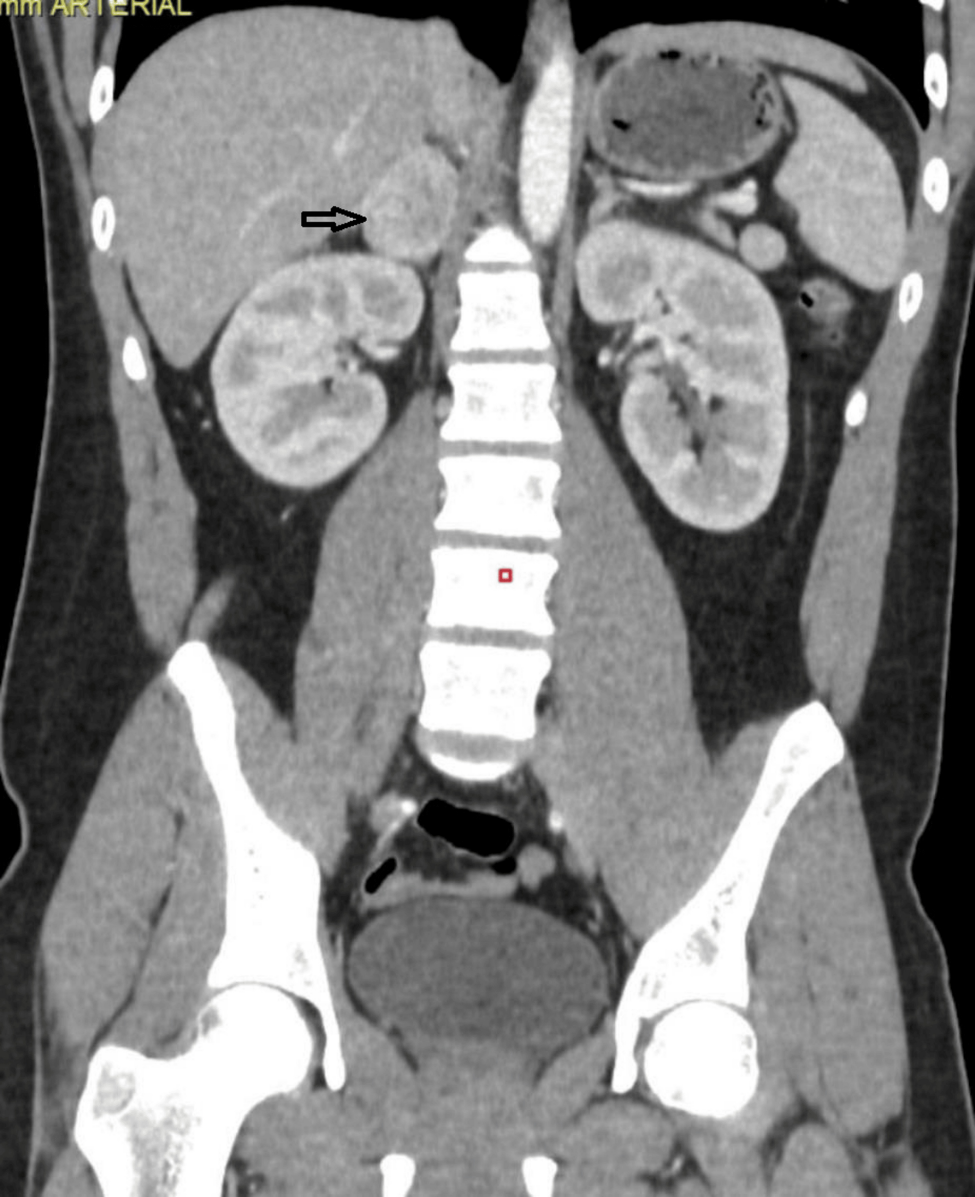
Contrast CT scan of the abdomen and pelvis cross coronal view showing a right adrenal tumor (measured as 4.2cm x 3.4cm x 4.8cm) (black arrow)

A contrast computed tomography (CT) scan of the abdomen and pelvis with an adrenal protocol was ordered, which showed a 4.2cm x 3.4cm x 4.8cm mass of the right adrenal gland (Figure 1). A diagnosis of a functional right pheochromocytoma was made, and the patient was referred to us for further surgical management.

The case was discussed at our multidisciplinary team meeting involving the intensivist, endocrinologist, and the surgeons. The patient and his parents were counseled, and informed consent was obtained from the parents. Preoperatively, the patient was managed on a competitive alpha antagonist, terazosin. No beta blockers were initiated as the patient did not develop reflex tachycardia during the course. The patient was also advised to eat a high-sodium diet with increased fluid intake while on the alpha blocker. 

**Figure 2:**
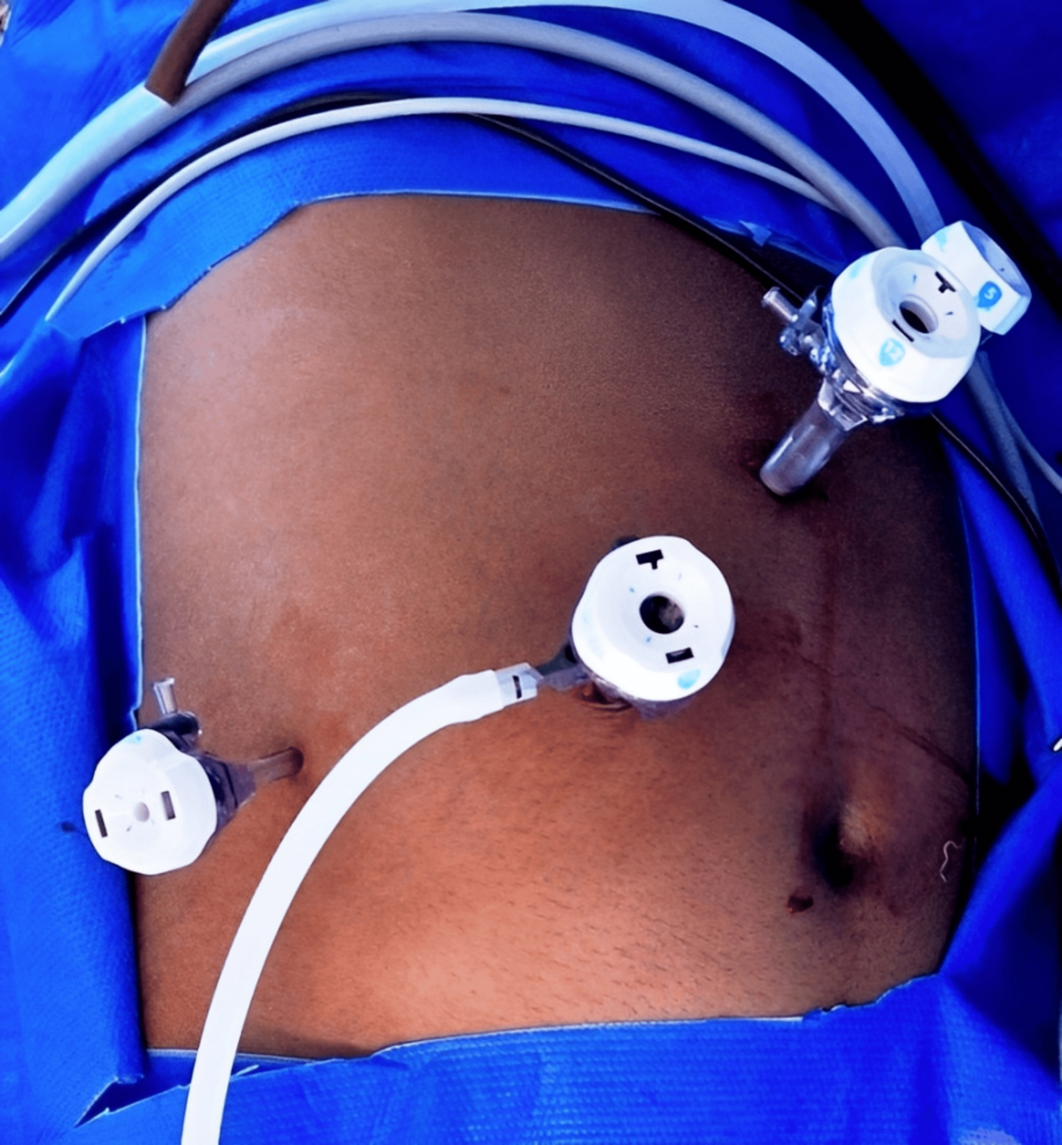
Photograph showing port placement for right trans-abdominal adrenalectomy

**Figure 3:**
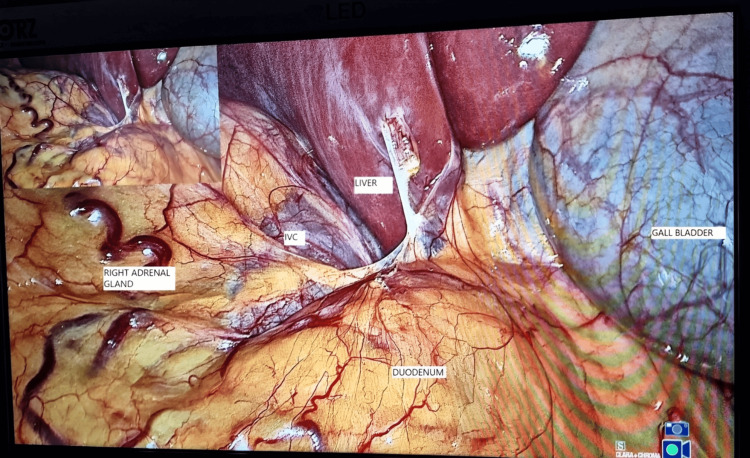
View on initial laparoscopy showing the position of important structures

Under general anesthesia with endotracheal intubation, the patient was placed in a left lateral position. A three-port technique was used; on initial laparoscopy (Figure 2), the right adrenal gland was noted to be covered by peritoneum/omentum, and the inferior vena cava (IVC) was noted just lateral and posterior to the gall bladder (Figure 3).

**Figure 4:**
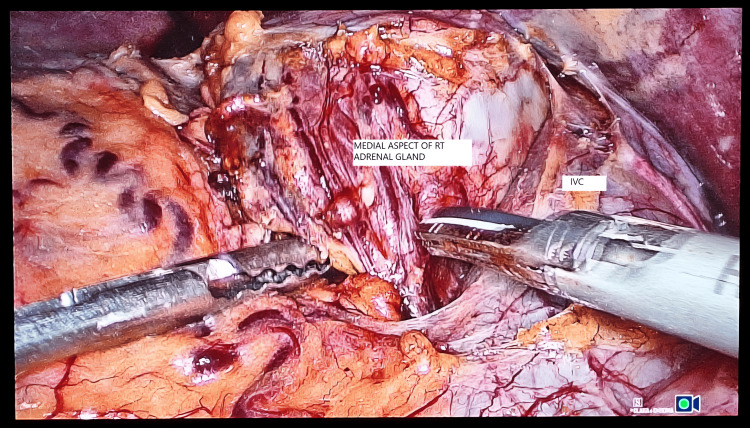
Intra-op picture demonstrating medial dissection along the right adrenal gland to expose the lateral aspect of the inferior vena cava

**Figure 5:**
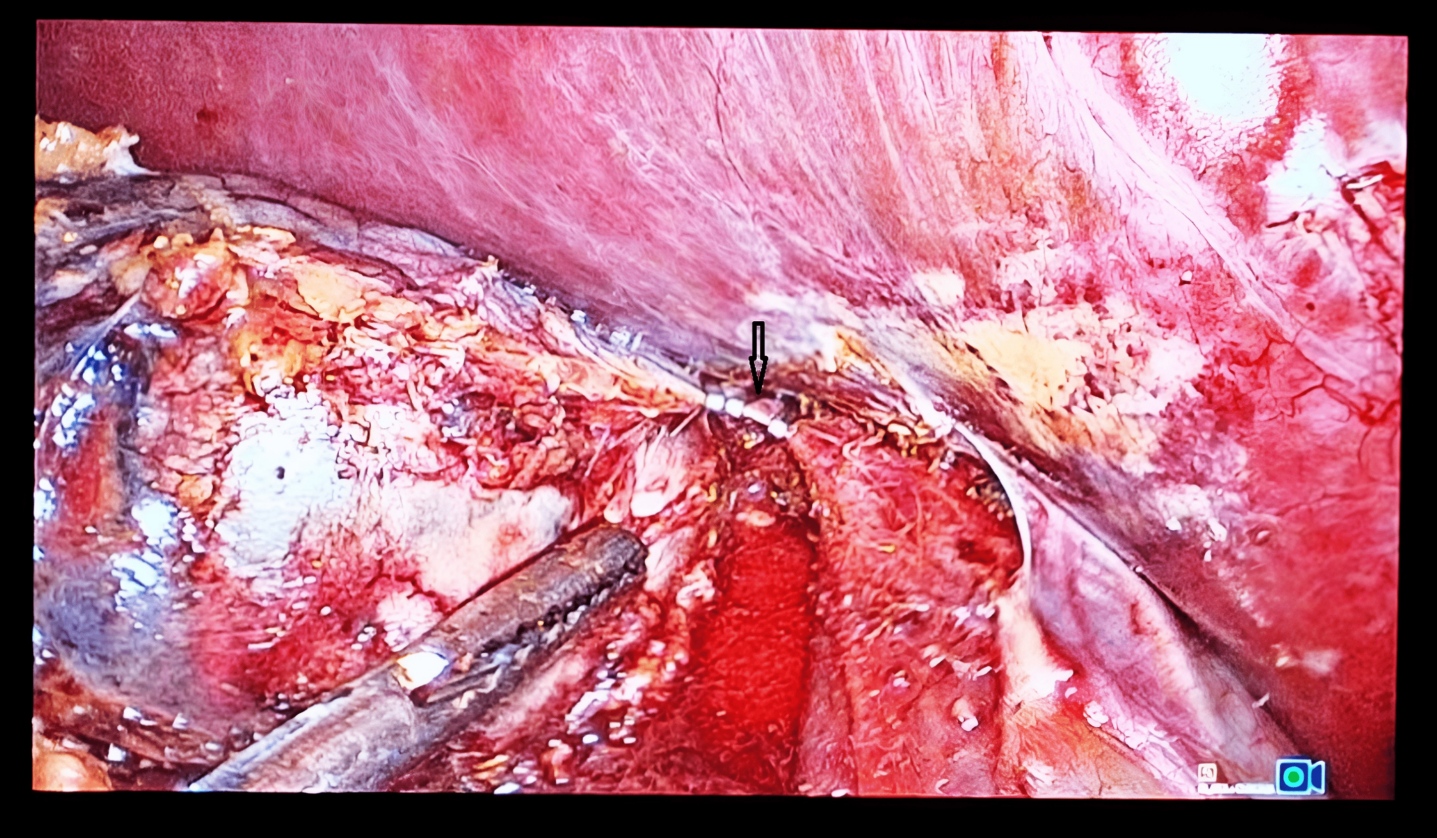
Intra-op photo showing the clipped right adrenal vein

Dissection starts by incising the peritoneum along the lateral aspect of the IVC to expose it (Figure 4). A meticulous non-handling of the adrenal gland was used to minimize intra-op catastrophes. Dissection is carried down along the lateral aspect of the IVC up to the superior border of the right renal vein and then continued posteriorly and inferiorly, safeguarding the renal artery or its branches. Dissection was then continued in a cephalad direction; the right adrenal vein was identified just lateral to the IVC, and it was then doubly clipped (Figure 5) and divided.

**Figure 6:**
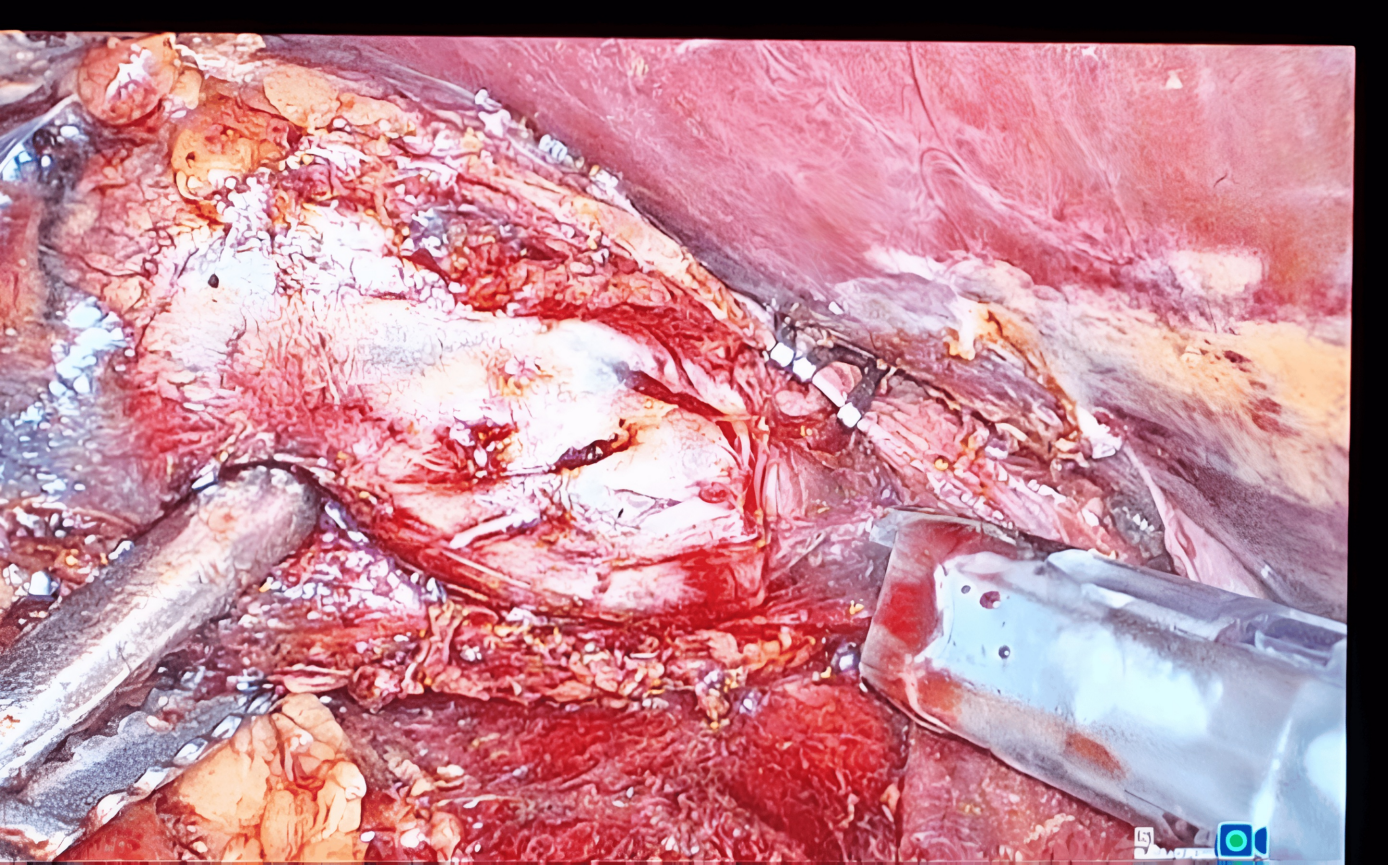
Intra-op photo showing clipping of branches of the right inferior phrenic artery

**Figure 7:**
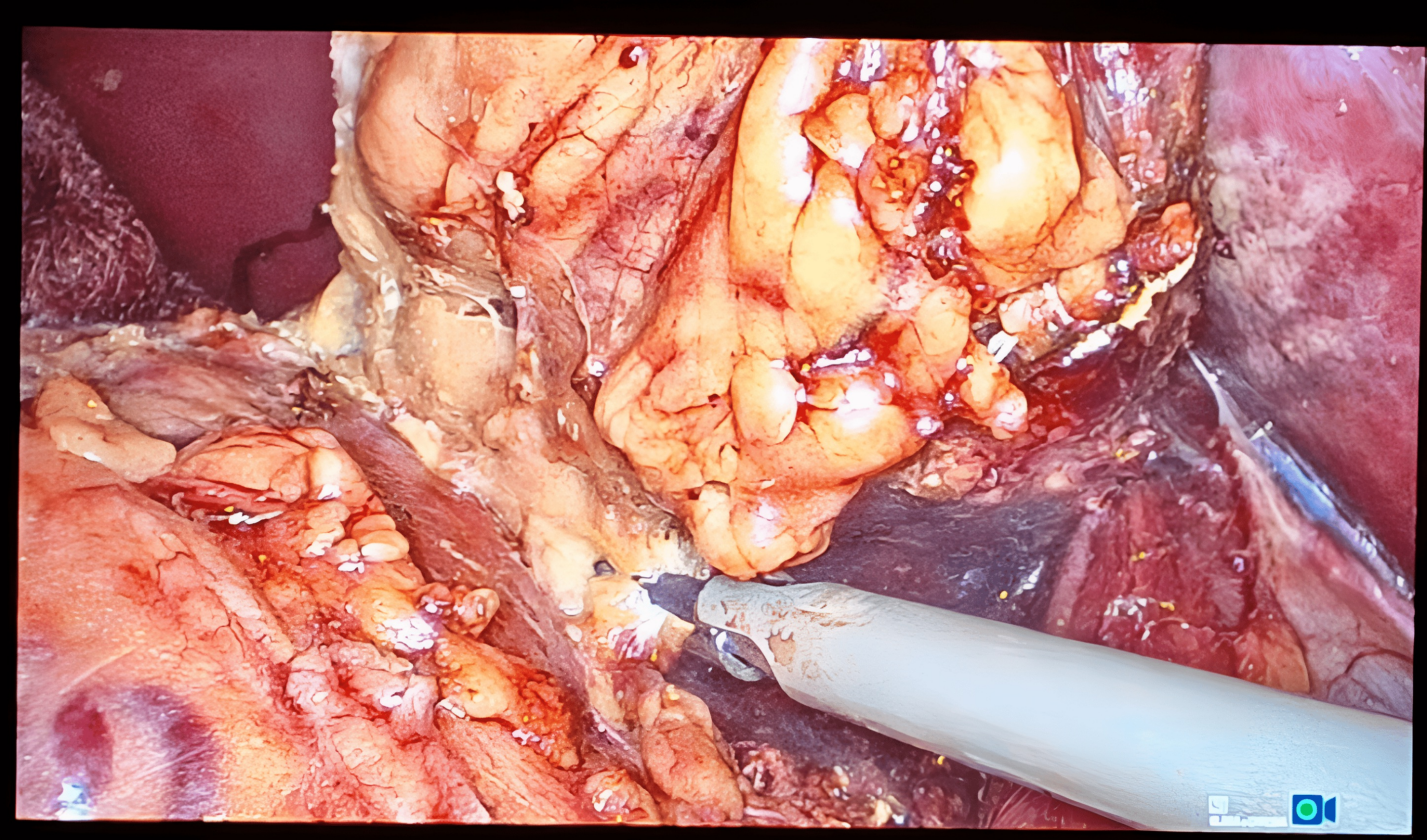
Intra-op photo showing final dissection of the right adrenal gland

The dissection was then carried superiorly, and branches of the inferior phrenic vessels were cauterized with the harmonic instrument (Figure 6). Careful dissection of the gland is performed to separate it from the IVC, where it is closely adhered to the IVC by gentle medial traction of the IVC. The lateral dissection is now performed, and right adrenalectomy was completed (Figure 7).

**Figure 8:**
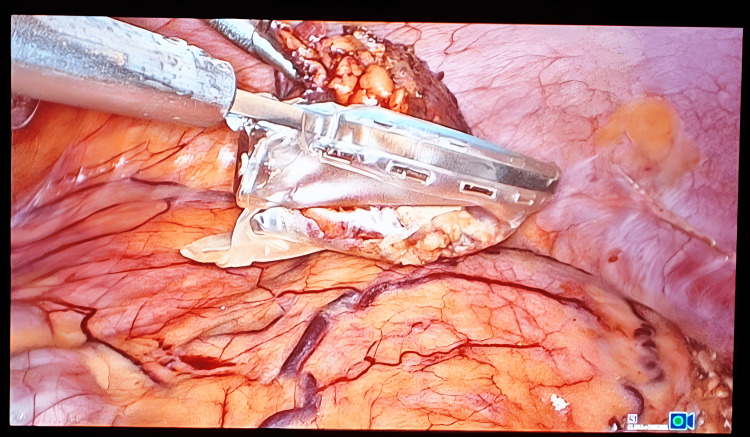
Intra-op photo demonstrating removal of the right adrenal gland by an endoscopic retrieval bag

**Figure 9:**
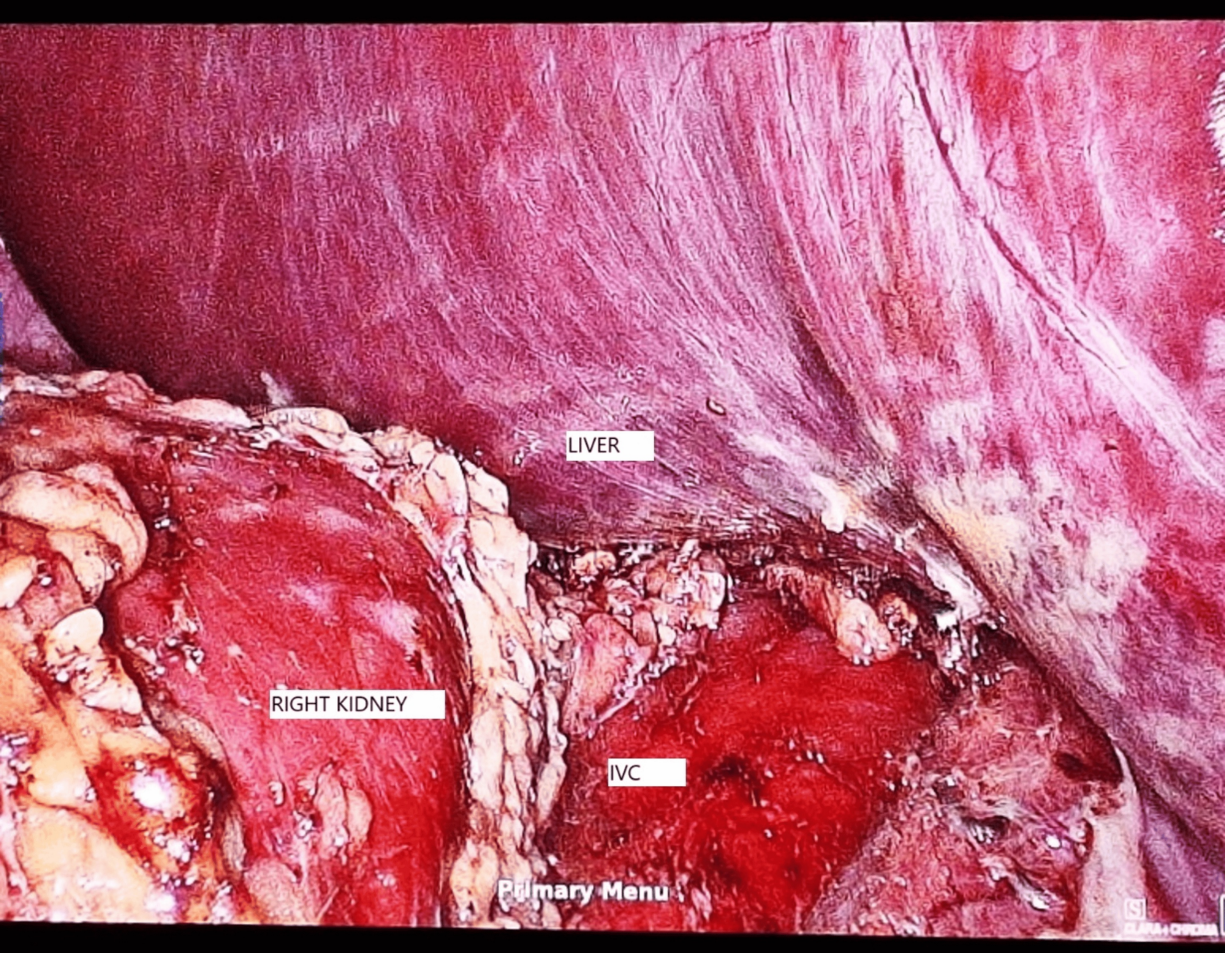
Intra-op photo showing the empty bed of right adrenal gland after removal

**Figure 10:**
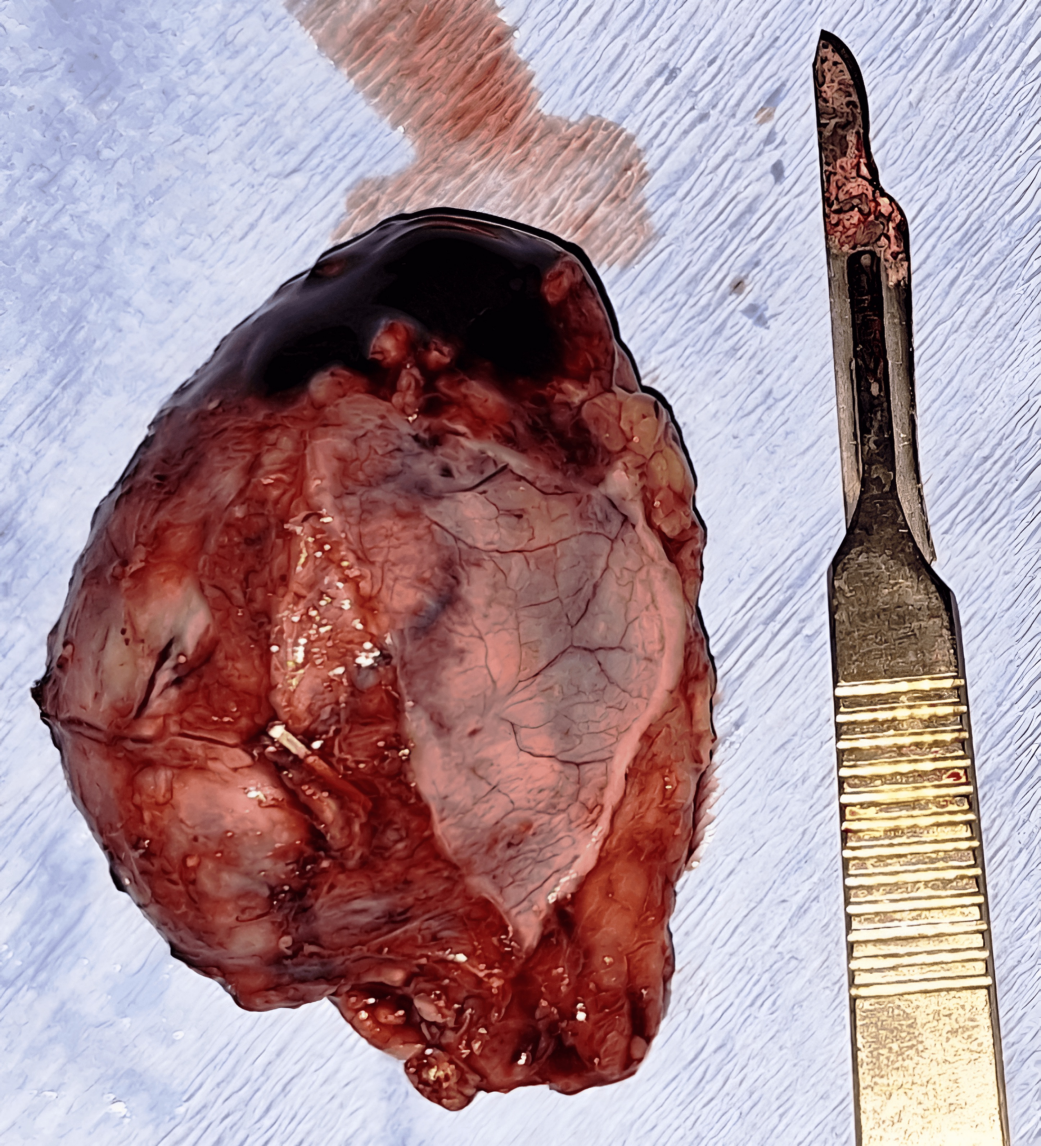
Postero-inferior view of the excised right adrenal gland

**Figure 11:**
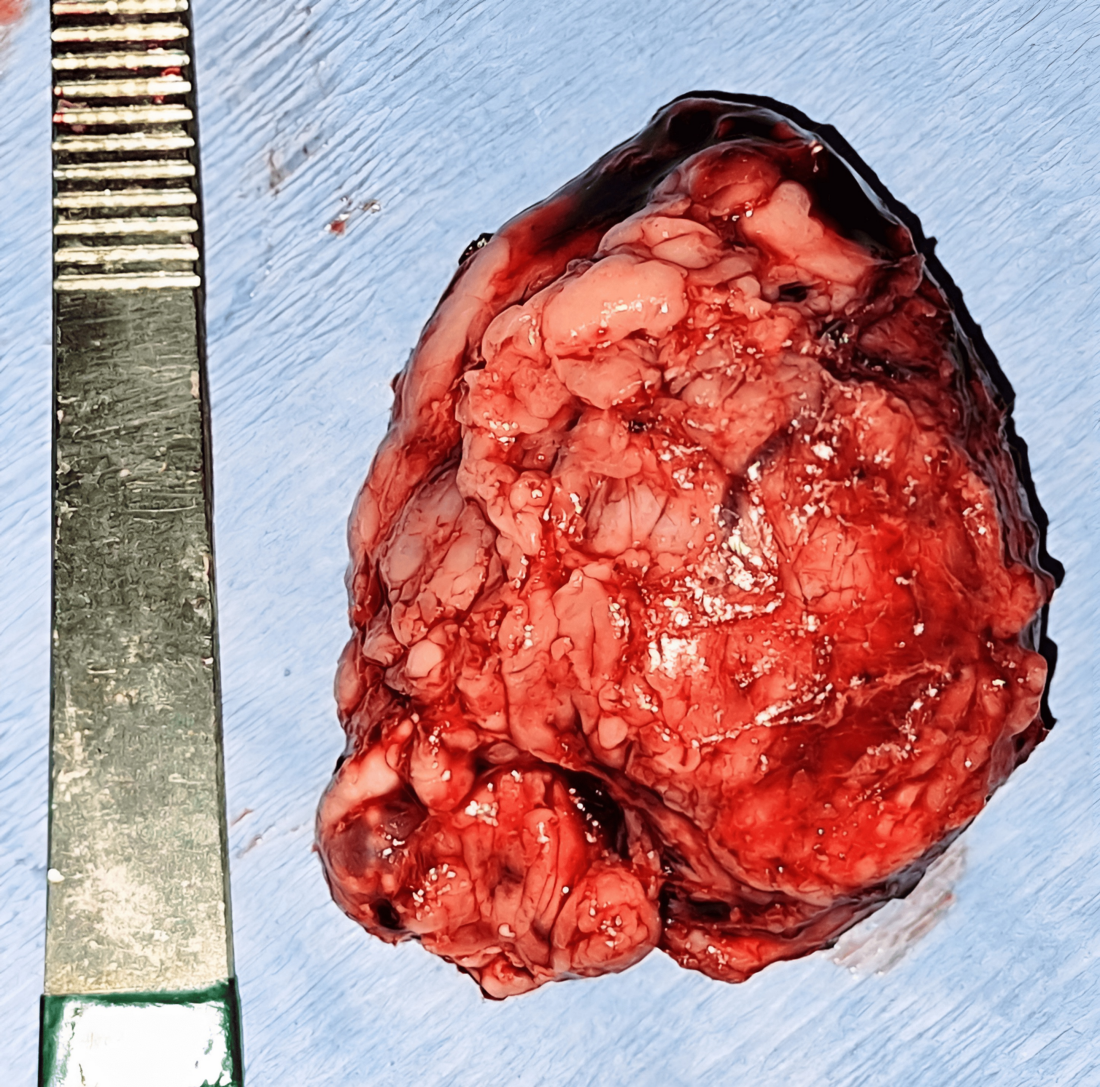
Antero-superior view of the excised right adrenal gland

The right adrenal gland was now removed with an endoscopic retrieval bag (Figure 8). The right adrenal bed (Figure 9) was inspected; hemostasis was achieved. Intraoperatively, the patient’s hemodynamic status was closely monitored and adjusted using fluid therapy and vasopressors when needed. The excised right adrenal gland measured as 6 cm x 5 cm, posterior-inferior view (Figure 10) and anterior superior view (Figure 11).

Post-operatively, the patient was admitted to a high-dependency unit and was weaned off vasopressors on day one post-op. After transfer to the surgical ward, the patient was discharged with satisfactory BP readings, not influenced by anti-hypertensives, on day two post-op. The patient was subsequently followed up in the outpatient surgical clinic, where he remained asymptomatic.

**Figure 12:**
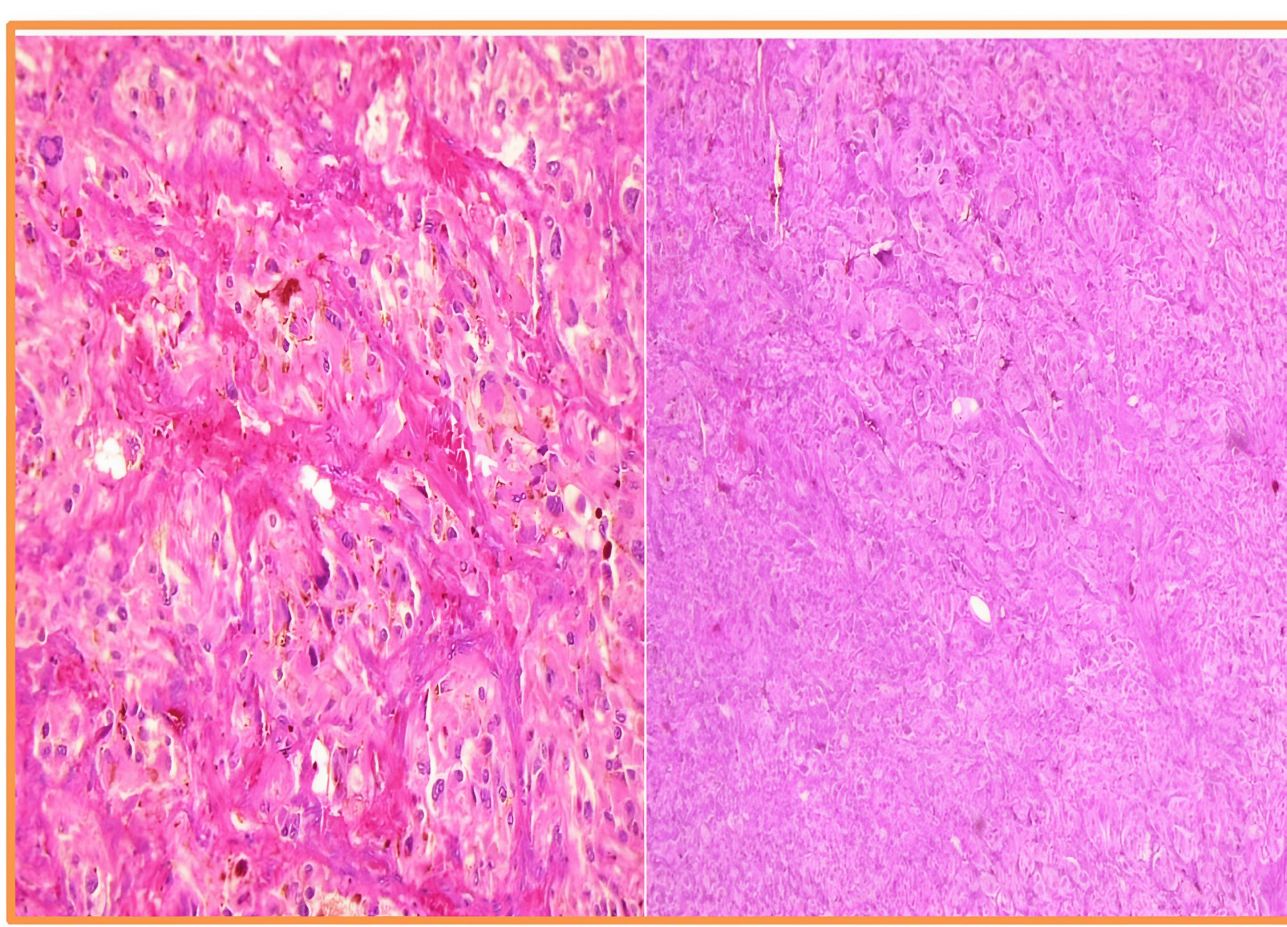
Histo-micrographs showing a well-circumscribed tumor of adrenal medulla consisting of large, polygonal, extensively vacuolated cells arranged in a nested and trabecular pattern. The cells show abundant granular cytoplasm with pigmented granules. The nuclei are round to oval and exhibit extensive variation in nuclear size with prominent nucleoli.

The final histopathological report confirms the diagnosis of pheochromocytoma of the adrenal gland with a scaled score (PASS score) 2 (Figure 12). At 15 months of follow-up, his BP log remained within normal limits and HbA1c returned to a normal value of 5.2%. 

## Discussion

Pheochromocytomas are rare tumors of the chromaffin cells of the adrenal medulla. In the adolescent population, it is a rare cause of secondary hypertension, occurring at a rate of 0.2 -0.6% of all subjects with hypertension [4]. In these young patients, the aetiology is more likely to be familial in origin than in adults, accounting for up to 80% of cases in patients under 18 years [5]. The typical familial syndromes associated with pheochromocytoma include multiple endocrine neoplasia type 2 (MEN 2), von Hippel-Lindau (VHL) syndrome, and neurofibromatosis type 1 (NF1). Knowing the above information, genetic testing should be considered in all cases of pheochromocytoma in the adolescent population. In addition to the classic syndromes, MEN 2, VHL, and NF1, germline succinate dehydrogenase gene mutations (SDHx) are included as part of the pheochromocytoma syndromes [6]. These syndromes are autosomal dominant and have varying penetrance. MEN 2 syndromes (caused by a mutation in the RET protooncogene), in particular, warrant evaluation of associated pathologies such as medullary thyroid carcinoma and hyperparathyroidism [7]. Before genetic testing, clinical and biochemical investigations can be done to facilitate targeted genetic testing, especially in resource-limited settings. Genetic testing is not available at our hospital, and our patients’ families also cannot afford to do the test privately. 

Immunohistochemistry (IHC), also not available in our hospital, can assist in the pathological diagnosis and molecular characterization of pheochromocytomas, especially in hereditary syndromes. Standard neuroendocrine markers include chromogranin A and synaptophysin, which are typically positive in pheochromocytomas, confirming neuroendocrine differentiation [8]. S100 protein highlights sustentacular cells, which are more prominent in benign lesions. A particularly valuable marker is SDHB; loss of SDHB expression on IHC is indicative of an underlying SDHx mutation. SDHB-negative tumors are associated with a higher risk of malignancy and recurrence, especially in familial cases. Additional markers such as Ki-67 (a proliferation index) can be used to assess tumor aggressiveness, although it is not diagnostic [9]. Thus, IHC not only confirms the diagnosis but also provides clues toward genetic etiology and prognosis.

Beyond IHC, the histopathological assessment of pheochromocytomas plays a critical role in evaluating their malignant potential. One of the most widely used scoring systems is the Pheochromocytoma of the Adrenal gland Scaled Score (PASS), introduced by Thompson in 2002 [10]. The PASS system evaluates the following histologic parameters that are thought to correlate with aggressive biological behavior: Large nests or diffuse growth, high cellularity, cellular monotony, tumor necrosis, increased mitotic figures, atypical mitoses, and vascular or capsular invasion.

Each criterion is given a weighted score, and a total score of ≥4 suggests a higher risk of malignancy or aggressive clinical behavior [10]. The PASS score alone does not guarantee clinical outcome and should be correlated with genetic and immunohistochemical scores mentioned previously.

In the evaluation of adolescent hypertension, secondary causes are more common than in adults and must be actively ruled out. Renal parenchymal disease (including glomerulonephritis, reflux nephropathy, and polycystic kidney disease) remains the most common cause of secondary hypertension in children, accounting for up to 60-70% of cases [11]. Renovascular hypertension, often due to fibromuscular dysplasia or renal artery stenosis, is another important differential diagnosis. Endocrine causes of hypertension, though less common, include Cushing’s syndrome, hyperthyroidism, hypothyroidism, primary hyperaldosteronism (Conn’s syndrome), and congenital adrenal hyperplasia. Pheochromocytoma and paragangliomas should be considered in any child with hypertension, palpitations, headaches, or sweating, especially when there is a family history of endocrine tumors or genetic syndromes [12]. Coarctation of the aorta, certain drugs and neurogenic causes such as increased intracranial pressure can also contribute to secondary hypertension [13].

The perioperative considerations remain similar to those of the adult undergoing adrenalectomy. Preoperatively, alpha blockers are given to reduce intraoperative hypertensive crises, beta blockers to prevent reflex tachycardia, and a high-sodium diet with increased fluid intake to prevent post-op hypotension by expanding the intravascular volume [6]. There are limited randomized controlled trials showing preferred pharmacology in the pediatric population, but multiple case studies, including ours, have shown success in using an analogous approach. 

The preferred surgical management is a laparoscopic adrenalectomy. Cortical sparing, to prevent cortisol deficiency, can be considered for bilateral pheochromocytomas, which are likely to occur in the familial syndromes, especially VHL2. However, the risk of recurrence and malignancy, especially in SDHx mutations, must be considered when choosing cortical sparing surgery [6]. Intra-operatively and postoperatively, invasive monitoring of blood pressures must be performed and adjusted accordingly by an experienced anesthesiologist. An experienced laparoscopic surgeon also plays an invaluable role in preventing perioperative complications. 

Because of the rarity of these tumors, data regarding prognosis are limited. Studies have shown that the survival rate of a benign pheochromocytoma is 100%, and for malignant disease, the 5, 10, and 15-year survival rates are 78%, 62%, and 38%, respectively [14]. Recurrence rates were found to be higher in familial cases, in particular VHL and SDH mutations, and as such, all young patients should be followed up for long-term surveillance. 

## Conclusions

This case emphasizes the infrequent presentation of pheochromocytoma in adolescents and demonstrates that, despite limited regional data, successful management is achievable even within a small Caribbean nation where existing research and case reports are sparse or absent. A multidisciplinary approach to diagnosis, perioperative care, and follow-up can lead to favorable outcomes and improved quality of life for affected patients.

However, the findings are limited by the single-case nature of this report, and outcomes may not be generalizable. There is also a need for more robust regional data, including long-term follow-up and postoperative quality of life assessments. Future considerations should include the development of national or regional registries, increased awareness, and targeted training to support timely diagnosis and evidence-based management of pheochromocytoma and other rare endocrine causes of hypertension in resource-limited settings.
